# Access Cavity Preparation and Localization of Root Canals Using Guides in 3D-Printed Teeth with Calcified Root Canals: An In Vitro CBCT Study

**DOI:** 10.3390/diagnostics13132215

**Published:** 2023-06-29

**Authors:** Kıvanç Kamburoğlu, Gül Sönmez, Cemre Koç, Funda Yılmaz, Osman Tunç, Abulfaz Isayev

**Affiliations:** 1 Department of Dentomaxillofacial Radiology, Faculty of Dentistry, Ankara University, 06560 Ankara, Turkey; 2 Department of Dentomaxillofacial Radiology, Faculty of Dentistry, Ada Kent University, 33010 Mersin, Turkey; 3 Department of Endodontics, Faculty of Dentistry, Adnan Menderes University, 09010 Aydın, Turkey; 4 Department of Endodontics, Faculty of Dentistry, Ankara University, 06500 Ankara, Turkey; 5 BTech Innovation, Teknokent METU, 06800 Ankara, Turkey; 6 School of Dental Medicine, Boston University, Boston, MA 02118, USA

**Keywords:** CBCT, 3D printing, guided endodontics, access cavity, pulp canal obliteration

## Abstract

Pulp canal obliteration (PCO) is a significant complication in endodontics that can occur due to various factors. Cone beam computed tomography (CBCT) is a useful diagnostic tool for identifying root canal anatomy and variations, and guided endodontics is emerging as an alternative treatment solution for teeth with partially or entirely obliterated pulpal canals. However, the accuracy of CBCT-guided 3D-printed guides on different materials and layer thicknesses is not well understood. Therefore, this study aimed to evaluate the accuracy of guides prepared using CBCT images on 3D-printed teeth with stereolithography (SLA) using three different materials and two different layer thicknesses. This study found that 3D-printed guides were accurate and reliable for accessing 3D-manufactured obliterated teeth and reaching the apical area. No significant differences in distance or angle measurements were found when different guide materials were used, suggesting that materials can be selected based on availability and cost. These findings contribute to the knowledge base regarding the effectiveness of 3D printing technology in guided endodontics and can help to identify the most suitable materials and techniques for this application.

## 1. Introduction

Pulp canal obliteration (PCO) is mostly characterized by the accumulation of calcified tissues within the root canal, and it can be caused by pulpal responses attributable to carious lesions, coronal restoration, vital pulp therapy procedures, dental traumatic injuries, and orthodontic treatment. Moreover, PCO has the potential to occur due to the apposition of secondary dentin in elderly patients [1,2,3,4]. According to the American Association of Endodontists, PCO is considered a significant clinical complication [5]. In particular, the endodontic treatment of severely calcified canals in symptomatic teeth or in teeth with radiographically detectable periapical disease is frequently challenging. In addition, the existence of variations, constricted canals, omitted curves, and curvatures may lead to perforations during preparation procedures [6,7]. These procedural complications might negatively affect the success of endodontic treatment by causing infections in inaccessible periapical tissue. In addition, even with the use of dental loupes or operation microscopes, access cavity preparation and canal orifice identification are challenging, and they can lead to excessive dental tissue removal, which is associated with poor long-term prognosis [8,9].

Cone beam computed tomography (CBCT) offers substantial clinical information for the visualization of the entire root canal anatomy and its variations in the axial, sagittal, and coronal planes. Thus, CBCT is frequently utilized in the diagnosis, treatment, and follow-up of difficult endodontic cases, such as PCO, with the advantages of ease of operation for endodontists, acceptable effective patient doses for endodontic patients, and high-definition tooth and pulp images with submillimeter accuracy [9,10]. Many of the endodontic signs obtained from the analysis of CBCT images were not correlated well with the corresponding intraoral radiographs. Therefore, 3D CBCT evaluations have strong potential to overcome the limitations of periapical intraoral radiography. The use of CBCT is recommended for extremely complex root canal anatomy and calcified canal evaluation with correct FOV settings, voxel size, mAs, appropriate kVp, and selection of the optimal parameters according to the ALADA concept (as low as diagnostically acceptable) [11]. 

Guided endodontics has become an alternative treatment solution for teeth with partially or entirely obliterated pulpal canals [12]. The advantages of three-dimensional (3D) planning and the creation of guided models for the localization of the root canal system is the basic concept of guided endodontics, which enables clinicians to prepare the access cavity and treat teeth with obliterated root canals [12,13]. Three-dimensional printing procedures involve digital data acquisition using an intraoral scanner and/or CBCT, data processing and design using computer software, and the manufacture of a template by printing. These template guides allow the calcified root canal to be created in a minimally invasive manner; thus, an optimal amount of the tooth structure can be preserved. This advanced technique offers successful prognosis with a lower risk of complications, along with procedural chair time efficacy [12,13,14,15].

In association with CBCT, improvements in 3D printing have made it possible to manufacture identical replicas of extracted human teeth that can be utilized for educational purposes with standardized phantom teeth [16,17]. In addition, this standardized model can be effectively used to compare endodontic instrumentations and evaluate obturation techniques [9,10,11,12]. To closely simulate the natural dental tissues and root canal system, these models can be produced with hydroxyapatite-based materials to emulate the root canal system and microstructure of natural dentin in a standardized manner [18].

The material and technique used for printing are important in terms of biocompatibility, sterilization procedures, and printing accuracy. Various techniques and materials on the market are utilized in the printing process of dental guides, and even in some published papers, 3D printing information is missing. Three-dimensional printing is a process in which multiple layers of material are added sequentially under computer control to create a 3D object. Stereolithography (SLA) is based on a photosensitive monomer resin that forms a polymer and solidifies when exposed to ultraviolet light. SLA is preferred for dental applications because it is cost-effective and surface features are easily obtained using resin. Resin best matches the surface characteristics for dental applications, with its advantages of sterilization, low cost, and shorter preparation time than powder and metal [19,20]. Unlike milling techniques, hollow and more complicated objects can be created with greater ease, and it is possible to print complex shapes and structures without excessive force. As the technique only uses what is required for production, it is desirable in situations where the overall weight and size of the raw material is an issue, such as in endodontic and dental applications. Some disadvantages are as follows: stereolithography is available only with light curable liquid polymers, extra steps are added in placing and removing support structures that may be used during the process, and the staircase effect is caused by layer-by-layer production. The STL file format is the most preferred one as it is accessible by and compatible with most software and medical 3D printers [19,20].

Considering the importance of possible differences in the technique, thickness, and material utilized in guided endodontic access cavity preparation, this study evaluated the accuracy of guides prepared using CBCT images on 3D-printed teeth with SLA using three different materials and two different layer thicknesses.

## 2. Materials and Methods

**Figure 1:**
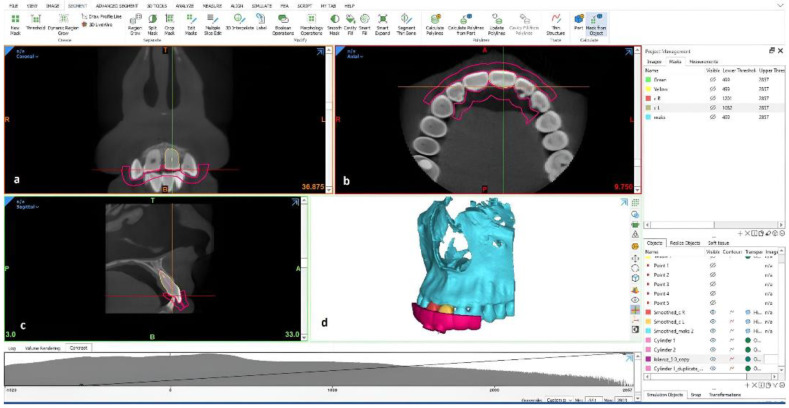
CBCT images showing 3D guide design using specific 3−matic modules and inbuilt tools. (**a**) Coronal plane. (**b**) Axial plane. (**c**) Sagittal plane. (**d**) 3D reconstruction of the maxilla and 3D guide.

Local ethical approval was obtained from the Ankara University Faculty of Dentistry Ethics Committee. A CBCT image of the maxillary left central tooth of a patient with a clearly visible oval root canal system, without calcification, was selected from our imaging archive, and Digital Imaging and Communications in Medicine files were exported and converted into Standard Tessellation Language (STL) files (segmentation images) using MIMICS^®^ Innovation Suite version 22.0 (Materialise NV, Leuven, Belgium, CE.0120 Certification). Using this software, artificial root calcification was performed on the apical third of the maxillary left central tooth in the design module (3-matic, Materialise). Guide designs were produced using the design tools in 3-matic. In the guide design, separate surface data were obtained for the relevant region by determining the guide seating surface. With the design commands to the surface data, a guide design compatible with the solid model and the tooth was rendered. The preproduction optimization processes of the designed guide model were optimized using Fix Wizard tools. Optimized files were exported in the STL file format to a 3D printer (Formlabs Form 2, Formlabs Inc., Somerville, MA, USA; Laser Specifications: EN 60825-1:2007 certified) and printed using biocompatible resin. The 3D models were washed, treated with a cure process, and detached from the support points. After determining the entry path and angle of the calcified root canals, 25 calcified 3D maxillary central incisor models were printed using clear resin as the printing material. In addition, a 3D maxillary model of the same patient with the empty socket of the left maxillary central incisor tooth was also duplicated (Figure 1).

To access 3D-manufactured obliterated teeth and reach the apical area, 3D guides were designed in the design module (3-matic), and the STL files were then exported to a 3D printer and printed using Dental SG, Gray Resin, and High Temp. In total, 25 guides were produced for five different guide groups of four teeth each, as follows: (1) Dental SG 50 µm, (2) Gray Resin 100 µm, (3) Gray Resin 50 µm, (4) High Temp 100 µm, and (5) High Temp 50 µm. Production was performed with a resin-based laser photopolymerization technique by using different thicknesses. After washing with isopropyl alcohol for 10 min, we cured the samples for 15 min at 45 degrees because they were small. According to company specifications, Formlabs resins are proprietary materials made of liquid photopolymers that were innovatively developed to work smoothly with all Formlabs SLA printers. Surgical Guide Resin is an autoclavable, biocompatible resin for applications including 3D-printing dental surgical guides for implant placement. Clear Resin is utilized for fluidics and mold making, optics, lighting, and any parts requiring translucency or showcasing internal features. High Temp Resin has a heat-deflection temperature (HDT) of 238 °C @ 0.45 MPa, which is the highest among the Formlabs resins. A Separate Resin Tank was required for High Temp Resin V1 and V2. The numbers 50 and 100 microns define the printing layer thickness. Each material does not have all layer thickness available, according to the company’s material development plan. The dental SG that we produced had only a 50-micron layer thickness parameter, the high-temperature resin had 50- and 100-micron layer thickness parameters, and the gray resin had all available 25-, 50-, and 100-micron layer thickness production parameters at the time when the present research was conducted.

Access to root canals and the removal of calcified parts of the root canals were conducted by a single operator (Endodontist—CK). A post drill was used for accessing the root canals and the removal of the calcified parts of the root canals in all experimental groups. A stopper on the drill was used to control depth. An endodontic motor in the gate mode (Xsmart; Dentsply Maillefer, Baillagues, Switzerland) was used. Fabricated 3D teeth were inserted into their respective 3D-manufactured sockets. For each guide, 3D-printed guides were first fitted onto teeth. Guides with a specific drill hole were designed to reach the apical foramen through the original root canal direction. A post space preparation drill with a diameter of 0.90 mm was used with pumping movements to penetrate the obliterated part of the root canal. An attempt was made to advance the drill into the canal without applying pressure. After every three pumping movements, the root canal was irrigated with physiological saline. This was progressed to 1 mm shorter than the working length, and then the root canal foramen was checked with a stainless-steel K-file. Thereafter, images of the teeth were obtained using Morita 3D Accuitomo 170^®^ (J Morita, Kyoto, Japan) under the following settings: 90 kVp, 5 mA, voxel size of 0.08 mm, 40 × 40 mm limited field of view (FOV), 1 mm thickness, and 1 mm interval. 

**Figure 2:**
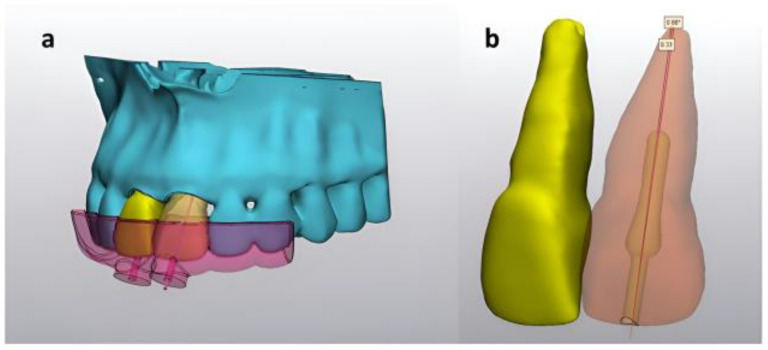
(**a**) Segmented maxilla and artificial maxillary central incisor with guide design. (**b**) Measurement of differences in the distance between the anatomical root apex and artificial root canal obliteration preparation on the sagittal plane (0.33 mm) and angle between the long axis of the tooth and canal obliteration preparation long axis (0.88°).

For each guide, two different measurements were obtained from postpreparation CBCT images using MIMICS^®^ Innovation Suite version 22.0 as follows: (1) distance from the anatomical root apex to the artificial root canal obliteration preparation in the sagittal plane and (2) angle between the long axis of the tooth and canal obliteration preparation long axis (Figure 2).

A one-sample *t*-test was used for descriptive analysis, and one-way ANOVA was used for statistical analysis. The significance level was *p* < 0.05.

## 3. Results

**Figure 3:**
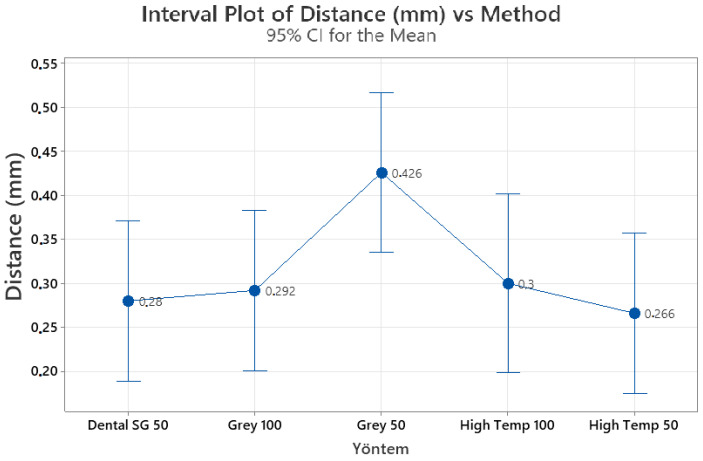
Mean differences in the distance between the anatomical root apex and root obliteration for different guide materials.

**Figure 4:**
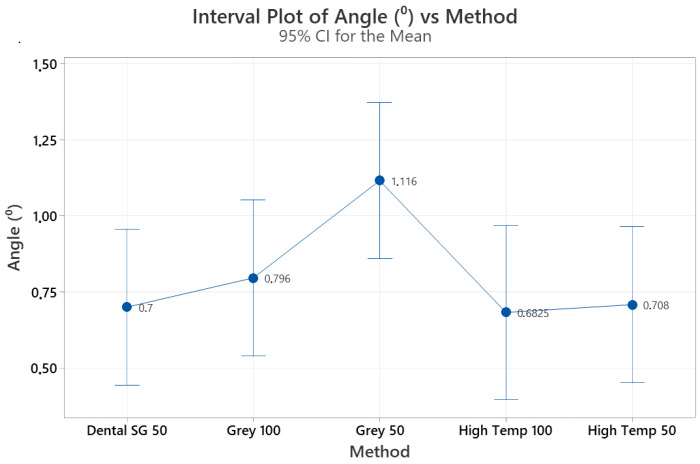
Mean angle differences measured for different guide materials.

Table 1 shows descriptive data regarding the angle and distance measurements. When differences in distance between the anatomical root apex and root obliteration were considered, no statistically significant differences were found among the 3D guides (*p* = 0.107). Table 2 shows the mean differences in length between the anatomical root apex and root obliteration for different guide materials. Figure 3 presents the mean distance differences measured for different guide materials and layer thicknesses. The mean differences were 0.28 ± 0.0721 mm for Dental SG 50 µm, 0.2920 ± 0.133 mm for Gray Resin 100 µm, 0.426 ± 0.0733 mm for Gray Resin 50 µm, 0.3 ± 0.082 mm for High Temp 100 µm, and 0.26 ± 0.106 mm for High Temp 50 µm. In addition, no difference was identified among the 3D guides regarding the angle between the long axis of the tooth and canal obliteration preparation long axis (*p* = 0.114). Table 3 shows the mean differences in this angle for different guide materials, and Figure 4 presents the mean angle differences for different guide materials. The mean differences were 0.70° ± 0.2053° for Dental SG 50 µm, 0.796° ± 0.378° for Gray Resin 100 µm, 1.1160° ± 0.2046° for Gray Resin 50 µm, 0.682° ± 0.261° for High Temp 100 µm, and 0.708° ± 0.281° for High Temp 50 µm.

## 4. Discussion

We compared the effects of the material type and layer thickness on the effectiveness of the 3D guides for the 3D printing of the calcified root canal system. The distance and angle measurements did not reveal any significant differences among the guides. Prior studies on 3D printing in endodontics were mainly case reports and preclinical investigations, which demonstrated promising outcomes and potential benefits, such as reducing the duration of the operation and the risk of procedural errors [2,19]. 

The material type, layer printing thickness, CBCT unit and workflow parameters, and practician experience may all affect the success of the guided treatment of calcified root canal systems both in vivo and in vitro. The resin materials chosen for the present research are standard, industrial, and dental guide resins from a specific company. Our production plans were developed and production process conducted according to the following considerations: Dental SG was chosen because it can be autoclaved and easily used in the dental field. High temp resin is resistant to high temperatures; therefore, it was taught that abrasion and peeling would be late due to high thermal stability, especially during touring; finally, gray resin was included as it is economical and readily available in comparison to others. In addition, layer thicknesses of 25, 50, and 100 microns affect the production resolution. The lower we go in resolution and detail, the longer the production time. Production in 1 h at 100 microns can increase to 6 h at 25 microns. Therefore, we chose the most routinely preferred parameters according to our daily experiences and pilot studies available at the time of this study. Now, the types of materials and their parameters have increased. 

CBCT offers dental and cros-sesctional images with submillimeter precision, which are crucial for producing accurate printed guides. In this study, small FOV CBCT images recommended for endodontic applications were used, along with an optimal voxel size. For determining the location, extent, and length of the calcified canal, optimal CBCT data are necessary. This information enables accurate and effective guidance for the root canal treatment of calcified canals by determining the shape of the root canal. In this study, we also employed virtual planning through a design process and prepared drill holes for guided endodontic treatment [19,20,21,22,23,24,25,26,27,28,29,30,31,32].

One of the clinical issues regarding the application of the guided access cavity preparation approach is the selection of optimum CBCT parameters to visualize the root canal anatomy, along with the selected materials and techniques used to construct 3D guides. The voxel is the smallest element of a CBCT image, and the voxel size is critical for image quality, scanning times, and patient radiation doses. Clinically, it is not possible to visualize the root canal on CBCT images obtained at a voxel size larger than the canal size. However, decreasing the voxel size might increase the patient radiation dose and image noise. Therefore, it is important to determine the most appropriate voxel size for a given CBCT system during the 3D printing process. In the present study, high bone and teeth tissue detail was achieved using parameters of 90 kVp, 5mA, voxel size of 0.08 mm, and 40 × 40 mm limited FOV, as suggested for endodontic purposes [19,20,21,22,23,24,25,26,27,28,29,30,31,32]. Dose concerns regarding the use of CBCT during routine dental and endodontic clinical practice is an ongoing discussion. In this context, authors suggest the use of a low-dose CBCT protocol over the standard one in endodontic special cases with a significantly lower radiation dose on Alderson Rando phantoms (The Phantom Laboratory, New York, NY, USA) and GR-200 A LiF thermoluminescent dosimeters (TLDs) in vitro. They found that acquisition produced the lowest organ dose (5.01 microSv) at the level of the esophagus with an image quality accurate enough for routine endodontic diagnostic needs. However, further research is essential to determine the versatility of the proposed low-dose protocol in the diagnosis of calcified canals and fabrication of 3D models for guided access cavity preparation or for other endodontic tasks [33].

One disadvantage of CBCT machines is their inability to provide clear soft tissue contrast, which can be significant in situations where soft tissue detail is critical. Consequently, merging the data from intraoral scanners with CBCT data can enhance image quality. This study did not incorporate the use of intraoral scanners and fused images, as they focus solely on hard tissue structures. It is important to select appropriate technical parameters, such as voxel size, kVp, mA, and software for CBCT imaging, to produce the high-quality radiographic images that are necessary for 3D printing. This study presented a novel protocol that utilized 3D reconstruction and SLA 3D printing technology to create endodontic guides for calcified root canals. The printed guides had a hole relief tolerance of 0.3 mm and a relief space of 0.3 mm, which were consistent across all guides presented [19,20,21,22,23,24,25,26,27,28,29,30,31,32].

In a previous investigation [34], the precision in using two distinct software applications for guided endodontic access (GEA) through digital workflow was compared in 3D-printed teeth meant to imitate PCO in vitro. Three-dimensionally printed incisors were produced with simulated PCO and installed on study arches. CBCT and 3D surface scans were matched and employed to plan and prepare GEA virtually by one operator using two different techniques: (1) CoDiagnostiX (CDX; Dental Wings) with 3D-printed templates and (2) Sicat Endo (SE; Sicat) with subtractive CAD/CAM-manufactured templates. To perform a comprehensive examination, the post-surgery CBCT and virtual planning data were overlaid. Accuracy was determined by computing the discrepancies between the planned and prepared cavities at the tip of the bur (three spatial dimensions, 3D vector, and angle), and the virtual planning effort was measured by the time and quantity of computer clicks. Each sample’s 95% confidence interval was determined, and SE achieved a 100% success rate in locating root canals for GEA in all 16 cases, compared with 94% for CDX in 15 out of 16 cases. SE produced less mean deviation at the tip of the bur regarding the distance in the labial–oral direction (0.12 mm), 3D vectors (0.35 mm), and angle (0.68°) than CDX (0.54 mm, 0.74 mm, and 1.57°, respectively; all *p* < 0.001). CDX necessitated less time for planning and effort for each four-tooth arch (10 min 50 s, 107 clicks) than SE (20 min 28 s, 341 clicks; *p* < 0.05). The presented methods facilitated quick drill path planning, a GEA procedure that can be performed with a high degree of certainty and accuracy, and accurate identification of root canals in teeth with PCO. Our research revealed similar precision for all material groups tested. Nonetheless, we solely employed the Mimic software and additive manufacturing technology, which implies that we did not assess diverse software options, in addition to additive and subtractive manufacturing techniques [34].

In another study [35], the effectiveness of computer-guided endodontic access cavity preparation was evaluated in terms of success rate and tooth substance removal. Thirty typodont teeth with root canals were used and divided into two groups: guided endodontics and control. After obtaining CBCT, virtual planning was conducted for access cavity preparation, and a template was created using 3D printing. In the control group, the conventional access technique was used for cavity preparation. Tooth substance removal was measured by comparing the weight of the teeth before and after preparation. The results showed that 93.3% of root canals were successfully located using guided endodontics compared with 100% using the conventional technique. Furthermore, significantly less tooth substance was removed in the study group than in the control group. The authors concluded that guided endodontics could preserve a significant amount of tooth substance in normally calcified teeth. However, this approach should be balanced against the risks of radiation exposure, perforation, higher costs, and more difficult debridement and visualization of the pulp chamber and root canals [35].

Prior research evaluated the accuracy of a preparation procedure planned for teeth with PCO using a guide rail concept based on a CBCT scan merged with an optical surface scan. Forty-eight teeth were mounted in acrylic blocks. To simulate remnants of an apical root canal, an apical canal preparation was created and used as the target for a drill path. A guide rail was created by merging the scanned blocks with a CBCT scan. The metal sleeve within the guide rail was used to create a pathway for the bur to penetrate the dentine. Two examiners measured the distance between the centers of the performed drill path and apical target. A maximum distance of 0.7 mm was defined based on the radius of the bur (0.6 mm) and that of a root canal visible on a radiograph (0.1 mm). The t-test was used for evaluation, and intraclass correlation coefficients were used to express intra and inter-examiner reproducibility. The mean distance between the drill path and target was significantly shorter than 0.7 mm, and the intra- and interexaminer agreement was excellent. This technique could potentially be an important tool for addressing partial or complete post-cataract surgery posterior capsule opacification (PCO) [36].

A recent clinical report described the root canal treatment of seven obliterated teeth along with using 3D guides manufactured using three different materials and CAD-CAM techniques: an extrusion-based 3D printer (FDM), a photopolymerization-based 3D printer (SLA), and a milling process. The authors concluded that guided endodontics with precise virtual planning could be considered as a safe and predictable approach. They emphasized that the use of CBCT was crucial in terms of the assessment of the obliteration extent through the root canal. Additionally, they stated that more research needs to be performed on different planning software, materials, and designs for both 3D guides and burs [37]. 

Various studies have been conducted in the past regarding the accuracy and dimensional precision of 3D printing, and their findings have highlighted that the type of 3D printer utilized can play a crucial role in determining the quality of the prints [37,38,39,40,41,42,43,44,45]. Specifically, one study that compared three types of printers concluded that the costly PolyJet system could produce prints with higher accuracy, as well as reduce the processing time, when compared with SLA and multitjet printing. Furthermore, the researchers suggested that the PolyJet and SLA 3D printing systems are viable options for use in dental clinical applications due to their overall accuracy. Several factors can impact print quality, including the guide production process, printer characteristics, layer height, and rendering speed, which may cause print failures or increase the print time when printed at high resolution. Endodontists have reported limited proficiency in using computer-aided design software, and the cost of 3D printing may pose a significant obstacle to routine endodontic practice [37,38,39,40,41,42,43,44,45]. Although commercial dental lab printers may be too expensive for most endodontic applications, recent studies have evaluated the accuracy and precision of stents produced using desktop 3D printers, including Form2, Form3, and Carbon, and an independent commercial laboratory desktop 3D printer. Among these printers, Form2 was found to have the least sensitive apical deviations and was used in the study [45]. 

Authors suggest that the use of guided endodontics in certain cases may pose challenges and limitations. For example, patients with limited mouth opening or who do not use aligners adequately may experience changes in tooth position, which can hinder the accuracy of the treatment. It is, therefore, important to carefully consider the suitability of guided endodontics on a case-by-case basis. Furthermore, to preserve the original tooth configuration and prevent the need for new restorations, it is recommended to avoid placing new restorations after treatment planning. In situations where root canal calcifications are severe and extend up to the apical third, it may be necessary to plan and produce two templates, as reported in the case study. By carefully considering the limitations and challenges associated with static-guided endodontics, clinicians can ensure that the treatment is accurate and effective [37,38,39,40,41,42,43,44,45,46].

This study utilized a drill with a diameter of 0.9 mm. Previous reports suggest that bur sizes ranging from 0.85–1.3 mm are suitable for guided endodontics. Given the narrowness of calcified canals, it is advised that clinicians use small-diameter canal instruments with careful, slow, and ample irrigation while working under an operating microscope. In cases where the guided endodontic process fails to achieve the original path of the root canal, a new CBCT scan may be necessary. However, it is important to note that the software used for manual design may affect the probability of errors. The use of artificial intelligence technology can enhance mesh matching features, but manual processes may result in deviations in the root canal and adversely affect case planning, as reported previously [37,38,39,40,41,42,43,44,45,46].

Printed teeth have recently gained significant popularity, mainly in dental and endodontic education, as well as in endodontic research due to inabilities in collecting a sufficient number of extracted teeth, and concerns regarding hygiene problems and cross-contamination. In addition, in dental research production, the utilization of printed teeth has facilitated the standardization of study groups [47,48]. Such 3D-printed teeth, created by using micro CT or cone beam computed tomography (CBCT) data from extracted teeth, have been used successfully in studies on root canal filling and root canal preparation previously [49,50]. Due to differences in the manufacturing process and the material used for printing, 3D-printed teeth can have different properties. The use of 3D-printed teeth is suggested for studies of root canal shape, but not for the investigation of root canal cleanliness or any changes in dentin (surface, hardness, composition, and structure) [48,49]. It has been reported that the physical and mechanical properties of 3D-printed teeth allow the transfer of results to natural human teeth. In addition, 3D-printed teeth may offer a good opportunity to create well-standardized teeth, even with complex anatomies, canal shapes, and sizes, enabling the creation of fully balanced experimental groups [51]. As can be seen, the validity and accuracy of the image segmentation and 3D guide creation methodology that we used to compare linear and angular measurements have been previously demonstrated. The concordance of CBCT measurements with actual dimensions validates the use of this methodological approach. However, in applying our results to clinical situations, it should be made clear that it is more difficult to utilize guides in real clinical conditions and in patients’ mouths. Finally, it is neither ethical nor practical to conduct such a study on real patients. Further studies, designed for the comparison of different imaging parameters, printing technologies and settings, along with various printing materials on cadavers with real teeth and surrounding tissues should be encouraged. Therefore, the effects of periodontal structures and real teeth on the drilling and aligning procedures can be better analyzed. 

Machines tailored solely for dental applications are few and further research is needed to assess their effectiveness for various dentistry-related tasks. Many additive machines still do not print to the accuracy or reproducibility required for certain dental applications. In cases where increased accuracy is needed, the speed of production usually decreases. As research and development continue, the use of 3D printing technology in endodontics will become more extensive in the future. The expiration of patents has led to decreased equipment prices, and many companies are producing accurate machines at a lower cost. The availability of affordable bench-top printers, trained staff, and financial support will promote the use of 3D printing in endodontics. Clinicians will have a more active role in planning, designing, and printing clinically useful guides. Dental schools should include 3D dental printing in their curricula as knowledge advances. Research on optimal CBCT image quality for 3D dental printing is necessary. As technology advances and costs decrease, specialists will be able to make informed decisions about incorporating 3D printing into clinical practice. The study we conducted on the treatment of calcified root canals showed encouraging outcomes for the utilization of 3D printing technology, provided that appropriate case selection and design processes are followed. The application of this technology has the potential to decrease operative time, minimize operator bias, and mitigate the likelihood of procedural errors.

## 5. Conclusions

Root canal treatment in teeth with calcified canals represents a great challenge in endodontics. Guided endodontics developed to overcome this situation are gaining popularity in clinical practice. Thus, a need to identify the optimal guides to be produced for this purpose has arisen. The three-dimensionally printed guides produced from the resin materials chosen for the present research (Dental SG 50 µm, Gray Resin 100 µm, Gray Resin 50 µm, High Temp 100 µm, and High Temp 50 µm) were found to be accurate and reliable for accessing 3D-manufactured obliterated teeth and reaching the apical area. No significant differences in distance or angle measurements were found when different guide materials were used, with an overall mean distance measurement of 0.3133 mm ± sd 0.1068 and standard error of the mean of 0.0218 and an overall mean angle measurement of 0.8054° ± sd 0.3004 and standard error of the mean of 0.0613. In line with the findings of our study, because guided access cavity preparation using five different guides had no differences regarding angle and distance measurements, materials can be selected based on material availability and cost.

## Figures and Tables

**Table 1:** Descriptive data for the angle and distance measurements.

Variable	N	Mean	SD	SEM	95% CI for μ
Angle (°)	25	0.8054	0.3004	0.0613	(0.6786–0.9323)
Distance (mm)	25	0.3133	0.1068	0.0218	(0.2682–0.3584)

SD, standard deviation; SEM, standard error of the mean; CI, confidence interval.

**Table 2:** Mean differences in the distance between the anatomical root apex and root obliteration for different guide materials.

Guide	N	Mean	SD	95% CI
Dental SG 50 µm	5	0.2800	0.0721	(0.1891–0.3709)
Gray Resin 100 µm	5	0.2920	0.1337	(0.2011–0.3829)
Gray Resin 50 µm	5	0.4260	0.0733	(0.3351–0.5169)
High Temp 100 µm	5	0.3000	0.0829	(0.1983–0.4017)
High Temp 50 µm	5	0.2660	0.1060	(0.1751–0.3569)

SD, standard deviation; CI, confidence interval.

**Table 3:** Mean differences in the angle between the long axis of the tooth and canal obliteration preparation long axis for different guide materials.

Guide	N	Mean	SD	95% CI
Dental SG 50 µm	5	0.7000	0.2053	(0.4433–0.9567)
Gray Resin 100 µm	5	0.796	0.378	(0.539–1.053)
Gray Resin 50 µm	5	1.1160	0.2046	(0.8593–1.3727)
High Temp 100 µm	5	0.682	0.261	(0.395–0.970)
High Temp 50 µm	5	0.708	0.281	(0.451–0.965)

SD, standard deviation; CI, confidence interval.

## Data Availability

Data are unavailable due to privacy and ethical restrictions.
